# Supplementary material for: Allelic decomposition and exact genotyping of highly polymorphic and structurally variant genes
Source: Nat Commun. 2018 Feb 26;9:828. doi: 10.1038/s41467-018-03273-1 (PMC5826927; doi:10.1038/s41467-018-03273-1)
Supplement: Supplementary file 1 — Supplementary Information [file 41467_2018_3273_MOESM1_ESM.pdf]

1 Coverage Normalization

By analyzing 96 samples sequenced using the PGRNseq v.2 capture reagent, we have observed that the depth of coverage usually follows the same shape across different samples, as illustrated in Supplementary Figure 1.

In order to characterize the shape for PGRNseq data of an arbitrary sample S, we first consider the PGRNseq data for NA19686 individual. It is known that the *CYP2D6* genotype of this individual consists of two reference *1 alleles. We introduce the function

$$B_s : \{1, 2, \ldots, |s|\} \to \mathbf{R},$$

where s and $|s|$ denote the gene of interest (in our case *CYP2D6*, *CYP2D7* or *CYP2D8*) and its respective length. The value of $B_s(i)$ equals to the sum of coverage depths of both chromosomal copies for i-th nucleotide of gene s in the reference sample (NA19686). Sequencing experiments generating data for S and the reference sample are not necessarily equivalent in terms of depth of coverage. Consequently, we need an appropriate rescaling of function B_s in order to obtain the function of reference coverage depth for the sequencing experiment of sample S. Analogously to B_s, we define this function as

$$R_s : \{1, 2, \ldots, |s|\} \to \mathbf{R}.$$

Intuitively, $R_s(i)$ represents a depth of coverage for i-th nucleotide of the reference sample sequenced under the same conditions as the sample S. As B_s and R_s follow the same shape we can estimate R_s as

$$R_s(i) = \eta \times B_s(i), \qquad \forall i \in \{1, 2, \ldots, |s|\}$$

where η is the ratio of sequencing depths of the two experiments.

In order to estimate η, we use B_s and depth of observed coverage function for sample S, here denoted as C_s and defined analogously to R_s and B_s. Using region q of stable copy number that is not involved in any structural variations we can estimate η as

$$\eta = \frac{C_s(q)}{B_s(q)},$$

where $C_s(q)$ and $R_s(q)$ are obtained by summing all values of $C_s(i)$ and $R_s(i)$, respectively, for any i falling into the region q. One of the regions from *CYP2D* locus having this property is the region of *CYP2D8* containing exons 4, 5 and 6. Using this region as q in the above formula leads to a proper estimate of η that is later used for computing the reference coverage depth function R_s for sample S. Note that above R_s and C_s are not necessarily identical due to the possible presence of structural variations in sample S. Example of rescaling is given in Supplementary Figure 1.

Finally, we introduce the function $\text{cn}_s(i)$, denoting the normalized copy number at loci i within the gene g, as:

$$\text{cn}_s(i) = 2 \times \frac{C_s(i)}{R_s(i)}.$$

Coefficient 2 in the above formula is included to account for both chromosomal copies present in the sample. Analogously, we introduce the function $\text{mutcn}(m)$, which denotes the estimated copy number of m. Note that for a mutation m the value of $\text{mutcn}(m)$ is obtained by normalizing the number of reads that include

m by the expected coverage of m's locus, which is upper bounded by the normalized copy number of the region that covers m's locus, since it is not necessary that all reads that are mapped to this locus include mutation m (as they may originate from other copies of the gene).

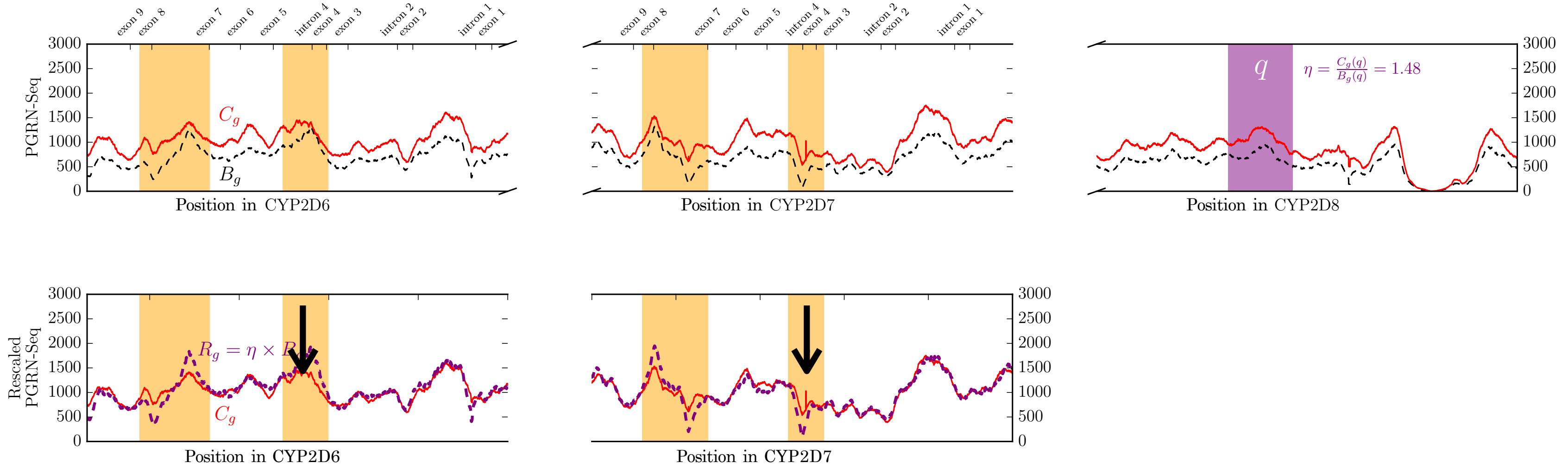

Supplementary Figure 1: Example of PGRNseq coverage rescaling for some sample S. The x axis represents the genomic loci, while y axis denotes the depth of coverage. The red line in the first row indicates the coverage of sample S, C_s, while the dashed black line indicates NA19686 coverage B_s. The purple dashed line in the second row indicates rescaled $R_s = \eta \times B_s$. Purple shading in the first row denotes the region q from *CYP2D8*. Identical regions (described in the section 2.5 of the main manuscript) are shaded in orange color.

(i) Regular case

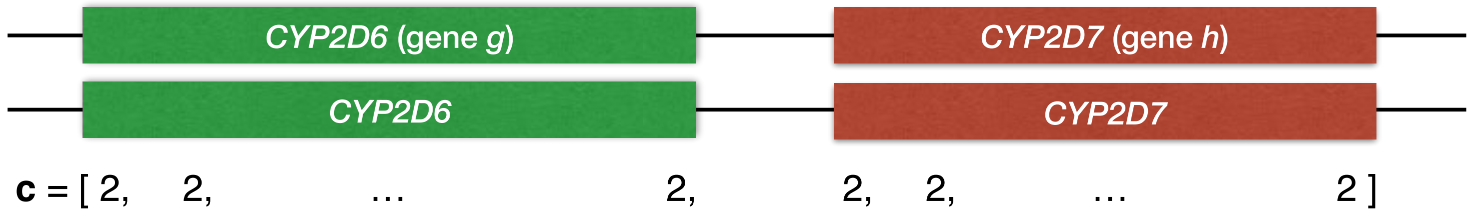

Supplementary Figure 2: PGRNseq coverage normalization for three *CYP2D6* gene arrangements. Coverage plots in the first row indicate the rescaled PGRNseq coverage, while plots in the second row indicate normalized copy number (i.e. C_s/R_s). Regions colored with red denote deletion of *CYP2D6*, while green-colored regions indicate gains (e.g. duplication). Identical regions (described in section 2.5 of the main manuscript) are shaded with orange color. Note the changes in vector **c**, which describes the observed coverage (and thus observed joint copy number structure) for each sample whose structure is depicted above it. In the last example (iii), the set of vectors **v** which most closely describe the vector **c** is given under the figure.

(i) Regular case: normal arrangement consisting of one copy of each of *CYP2D6* and *CYP2D7* on both chromosomes.

(ii) Deletion

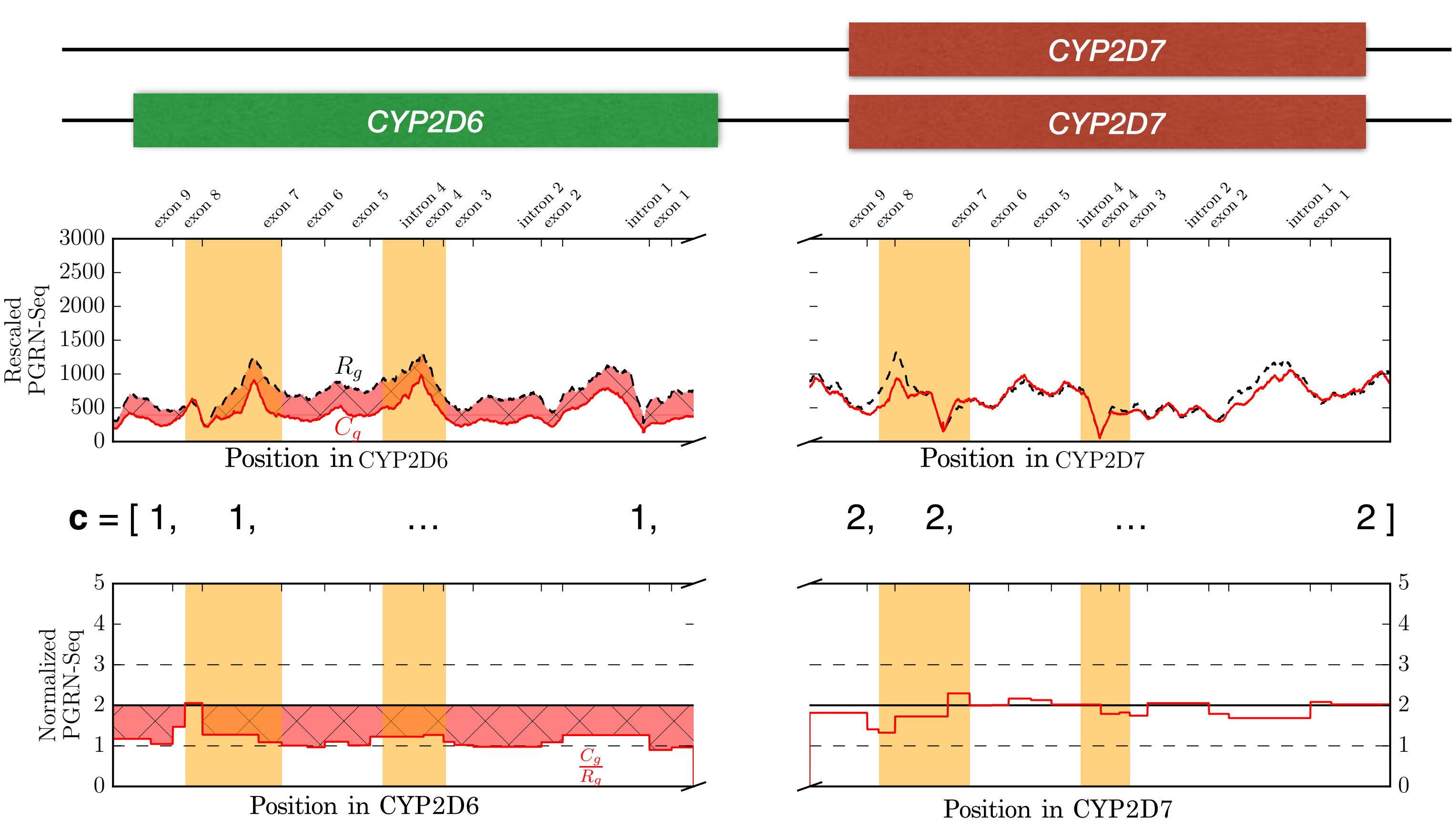

(ii) Deletion: *CYP2D6* deletion on one chromosomal copy.

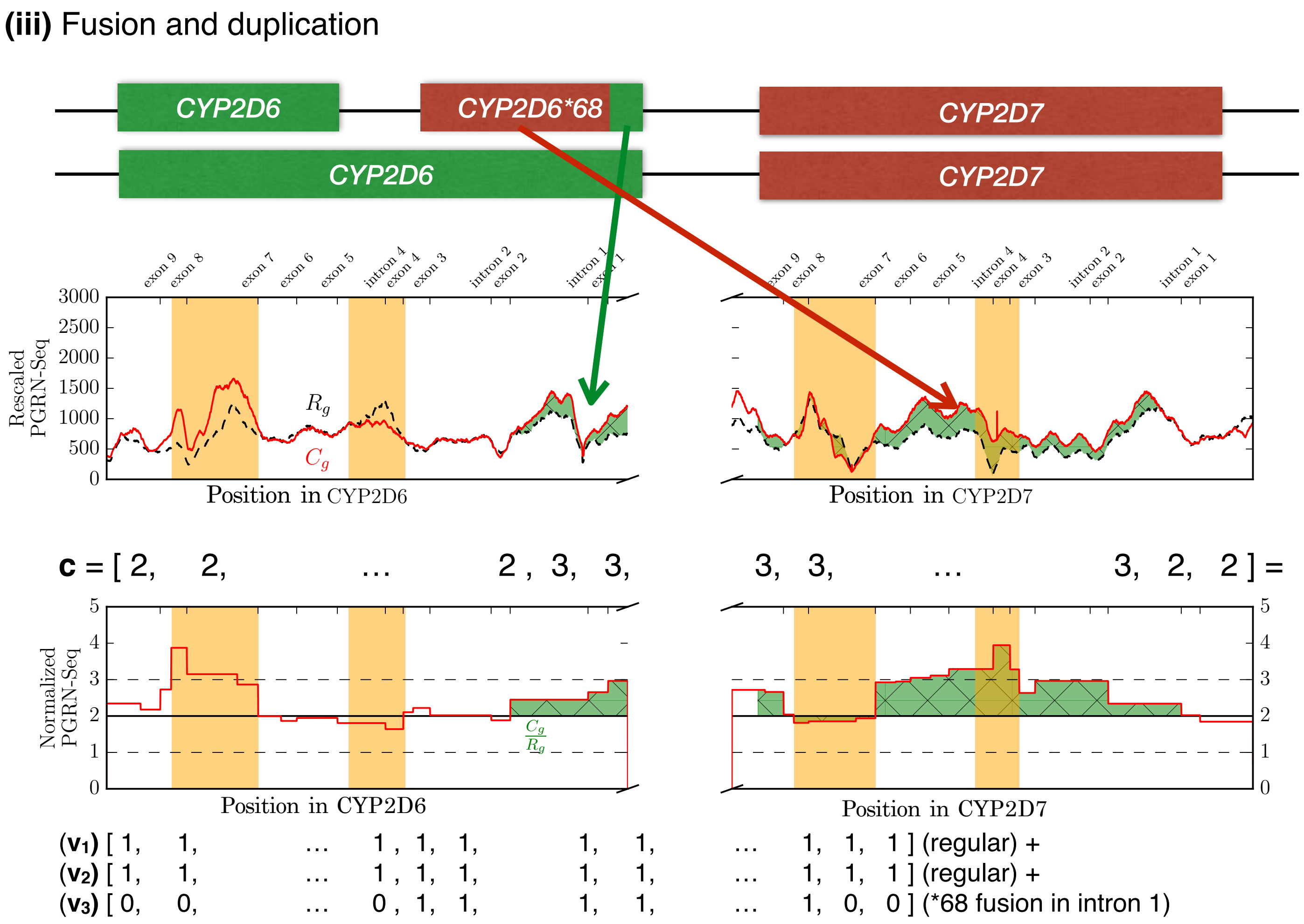

(iii) Fusion and duplication: one copy of *CYP2D6*1* accompanied by *CYP2D7/2D6* fusion (*68 allele) with the breakpoint in intron 1 on one chromosomal copy.

2 Complexity

In this section we show that Copy Number Estimation Problem (CNEP, section 2.2 in the main manuscript) and Major Star-Allele Identification Problem (MSAIP, section 2.3) are NP-hard. We prove these claims by reducing the Closest Vector Problem (CVP), introduced in [1], to both CNEP and MSAIP.

Problem 1 (CVP)**.** *Given an input consisting of: (i) a collection* $G = \{\mathbf{g}_1, \mathbf{g}_2, \ldots, \mathbf{g}_k\}$ *of binary vectors in* $\{0,1\}^d$*; (ii) a vector* $\mathbf{x}$ *in* $\mathbb{Z}_+^d$*, where* $\mathbb{Z}_+$ *denotes the set of non-negative integers; and (iii) a constant* $C \in \mathbb{Z}_+ \cup \{\infty\}$*, find a vector* $\mathbf{y}$ *among all vectors* $G\mathbf{u} = \sum_{j=1}^{k} u_j \mathbf{g}_j$ *with* $u_j \in \mathbb{Z}_+$ *for* $j \in \{1, 2, \ldots, k\}$*, minimizing* $\|\mathbf{x} - \mathbf{y}\|_1$ *while satisfying* $\|\mathbf{x} - \mathbf{y}\|_\infty \leq C$*. No solutions should be reported if no* $\mathbf{b}$ *satisfying* $\|\mathbf{x} - \mathbf{y}\|_\infty \leq C$ *exists.*

This problem is proven to be NP-hard, even with $C = \infty$ [1]. Here we provide a polynomial-time reduction from CVP to CNEP that directly implies NP-hardness of CNEP.

Theorem 1. *CNEP is NP-hard.*

Proof. Let us show that CVP is polynomial-time reducible to CNEP. We start our proof by assuming that an arbitrary instance of CVP with $C = \infty$ is given. As we are interested in an arbitrary non-negative integer linear combinations of vectors from G we may obviously assume that no two of vectors in G are equal. Provided that the elements in G are distinct binary vectors and that components of vector $\mathbf{a}$ are non-negative, we can construct an instance of CNEP where:

- $V = \{\mathbf{v}_1, \mathbf{v}_2, \ldots, \mathbf{v}_k\}$ and $\mathbf{v}_i = \mathbf{g}_i$ for $i \in \{1, 2, \ldots, k\}$; and
- $\mathbf{cn} = \mathbf{x}$.

Now, it is self-evident that solving the constructed instance of CNEP is equivalent to solving the given instance of CVP, hence the existence of polynomial time algorithm for CNEP would imply the existence of polynomial time algorithm for CVP. Since the above reduction is obviously done in the polynomial time, our proof is completed. □

Theorem 2. *MSAIP is NP-hard.*

Proof. In this proof we provide reduction from special instance of CVP with target vector $\mathbf{x}$ consisting solely of ones (the authors in [1] also prove that this particular instance of CVP is NP-complete).

Assume that we are given an instance of CVP with $\mathbf{x} = [1, 1, \ldots, 1]^T$, $C = \infty$ and set G. We reduce this problem to an instance of MSAIP where:

- $M = \{m_1, m_2, \ldots, m_{2d+1}\}$;
- $A = \{a_1, a_2, \ldots, a_{k+1}\}$ and mutation profile of a_i for $i \in \{1, 2 \ldots, k\}$ equals $[g_{i1}, g_{i2}, \ldots, g_{id}, 0, 0, \ldots, 0]$, whereas mutation profile of a_{k+1} equals $[1, 1, \ldots, 1]$. All of the mutation profiles vectors defined here are of length $2d + 1$ and, for each of them, its i-th coordinate encodes for the presence (=1) or absence (=0) of mutation m_i from the set of gene-disrupting mutations of a_i;
- $\mathrm{mutcn}(m_i) = 2$ for $i \in \{1, 2, \ldots, d\}$ and $\mathrm{mutcn}(m_i) = 1$ for $i \in \{d + 1, d + 2, \ldots, 2d + 1\}$;

and the objective is to find a set $\{p_1, p_2, \ldots, p_k, p_{k+1}\}$ of non-negative integers such that the sum of absolute values of components of vector $\mathbf{t}$ defined as

$$\mathbf{t} = \begin{bmatrix} g_{11} & g_{21} & \cdots & g_{k1} & 1 \\ g_{12} & g_{22} & \cdots & g_{k2} & 1 \\ \vdots & \vdots & \ldots & \vdots & \vdots \\ g_{1d} & g_{2d} & \cdots & g_{kd} & 1 \\ 0 & 0 & \ldots & 0 & 1 \\ 0 & 0 & \ldots & 0 & 1 \\ \vdots & \vdots & \vdots & \vdots & \vdots \\ 0 & 0 & 0 & 0 & 1 \end{bmatrix} \begin{bmatrix} p_1 \\ p_2 \\ \vdots \\ p_k \\ p_{k+1} \end{bmatrix} - \begin{bmatrix} 2 \\ 2 \\ \vdots \\ 2 \\ 1 \\ 1 \\ \vdots \\ 1 \end{bmatrix}_{(2d+1)\times 1}$$

is minimized. In the above equation, the last vector on right-hand side consists of d twos followed by $(d+1)$ ones (where d is the dimensionality of $\mathbf{x}$). We also add constraints related to the sum of p_i's that must be at least 1 as described in the definition of MSAIP. Below we prove that solving this instance of MSAIP suffices to find all optimal solutions of the given CVP. In order to prove this, we first define

$$y_i = \sum_{j=1}^{k} g_{ji} p_j.$$

Our goal is to find $\{p_1, p_2, \ldots, p_k, p_{k+1}\}$ such that

$$L_{\{p_1, p_2, \ldots, p_k, p_{k+1}\}} = \sum_{i=1}^{d} |y_i + p_{k+1} - 2| + (d+1)\,|p_{k+1} - 1|.$$

is minimized.

We claim that in any optimal solution we must have $p_{k+1} = 1$. In order to prove this claim, we first observe that changing the value of p_{k+1} by 1 (i.e. either increasing or decreasing it by 1) changes each summand in $\sum_{i=1}^{d} |y_i + p_{k+1} - 2|$ by at most 1, or in other words:

$$|y_i + (p_{k+1} \pm 1) - 2| - 1 \leq |y_i + p_{k+1} - 2| \leq |y_i + (p_{k+1} \pm 1) - 2| + 1.$$

Now consider an arbitrary $\{p_1, p_2, \ldots, p_k, p_{k+1}\}$. If $p_{k+1} = 0$ then considering $\{p_1, p_2, \ldots, p_k, 1\}$ (i.e. changing p_{k+1} to 1) we have

$$\begin{aligned} L\{p_1, p_2, \ldots, p_k, 1\} &= \sum_{i=1}^{d} |y_i + 1 - 2| \\ &\leq \sum_{i=1}^{d} (|y_i + 0 - 2| + 1) \\ &= \sum_{i=1}^{d} |y_i + 0 - 2| + (d+1) - 1 \\ &= L\{p_1, p_2, \ldots, p_k, 0\} - 1 \\ &< L\{p_1, p_2, \ldots, p_k, 0\}. \end{aligned}$$

Similarly, considering an arbitrary $\{p_1, p_2, \ldots, p_k, p_{k+1}\}$ where $p_{k+1} > 1$. By using $\{p_1, p_2, \ldots, p_k, p_{k+1} - 1\}$ (i.e. decreasing p_{k+1} by 1) we have

$$\begin{aligned}
L\{p_1, p_2, \ldots, p_k, p_{k+1}\} &= \sum_{i=1}^{d} |y_i + p_{k+1} - 2| + (d+1)(p_{k+1} - 1) \\
&\geq \sum_{i=1}^{d} (|y_i + p_{k+1} - 1 - 2| - 1) + (d+1)(p_{k+1} - 1) \\
&= \sum_{i=1}^{d} |y_i + (p_{k+1} - 1) - 2| + (d+1)\left[(p_{k+1} - 1) - 1\right] + 1 \\
&= L\{p_1, p_2, \ldots, p_k, p_{k+1} - 1\} + 1,
\end{aligned}$$

implying

$$L\{p_1, p_2, \ldots, p_k, p_{k+1} - 1\} \leq L\{p_1, p_2, \ldots, p_k, p_{k+1}\} - 1 < L\{p_1, p_2, \ldots, p_k, p_{k+1}\}.$$

From the above inequalities we conclude that for $p_{k+1} \neq 1$, the value of $L_{\{p_1, p_2, \ldots, p_k, p_{k+1}\}}$ can always be decreased by making appropriate update to p_{k+1} and this directly implies that in any optimal solution we must have $p_{k+1} = 1$. Furthermore, this also implies that the constraint on the sum of p_i's in MSAIP is always satisfied and consequently our optimization problem is essentially equivalent to finding $\{p_1, p_2, \ldots, p_k\}$ such that

$$\sum_{i=1}^{d} |y_i - 1|$$

attains its minimal value and no other constraint, except that they are non-negative integers, is imposed on $p_1, \ldots, p_k$. However, the last problem is exactly the instance of CVP given at the beginning of this proof. Since reduction constructed above is obviously polynomial this completes our proof that MSAIP is NP-hard. □

3 Results

3.1 Dataset (1): *CYP2D6* on PGRNseq v.2 data

Sample	Family	Predictions			Validation	Comments
		Aldy	Astrolabe	Cypiripi		
**HG00421**	SH007/F	✓ (*2/*10+*10)	✗ (*2/*10)	✗ (*65/*65+*65)	*2/*10$\times N$	Duplication
**HG00422**	SH007/M	✓ (*2/*10)	✓ (*2/*10)	✗ (*65/*65)	*2/*10	
**HG00423**	SH007/C	✓ (*10/*10+*10)	✗ (*10/*10)	✓ (*10/*10+*10)	*10/*10$\times N$	Duplication
**HG00463**	SH021/F	✓ (*36+*10/*36+*10)	✗ (*10/*10)	✗ (*10/*10+*10)	*36+*10/*36+*10	Duplication with fusion (*36)
**HG00464**	SH021/M	✓ (*1/*36+*10)	✗ (*1/*10)	✗ (*1/*10+*10)	*1/*36+*10	Duplication with fusion (*36)
**HG00465**	SH021/C	✓ (*36+*10/*36+*10)	✗ (*10/*10)	✗ (*10/*10+*10)	*36+*10/*36+*10	Duplication with fusion (*36)
**HG00592**	SH057/F	✓ (*1/*10)	✓ (*1/*10)	✓ (*1/*10)	*1/*10	
**HG00593**	SH057/M	✓ (*2/*36+*10)	✗ (*2/*10)	✗ (*39/*65+*65)	*2/*36+*10	Duplication with fusion (*36)
**HG00594**	SH057/C	✓ (*1/*2)	✓ (*1/*2)	✓ (*1/*2 or *34/*39)	*1/*2	
**HG01060**	PR14/F	✓ (*1/*41)	✓ (*1/*41)	✗ (*1/*2 or *34/*39)	*1/*41	
**HG01061**	PR14/M	✓ (*1/*4)	✓ (*1/*4)	✓ (*1/*4)	*1/*4	
**HG01062**	PR14/C	✓ (*1/*4)	✓ (*1/*4)	✓ (*1/*4)	*1/*4	
**HG01190**	PR40/F	✓ (*4/*68)	✗ (*4/*4)	✗ (*5/*4)	*68+*4/*5	Fusion (*68)
**HG01191**	PR40/M	✓ (*2/*41)	✓ (*2/*41)	✗ (*2/*2)	*2/*41	
**HG01192**	PR40/C	✓ (*5/*41)	✗ (*41/*41)	✗ (*5/*2)	*5/*41	Deletion (*5)
**HG01979**	PEL027/F	✓ (*2/*68+*4)	✗ (*2/*4)	✗ (*4/*65)	*2/*68+*4	Fusion (*68)
**HG01980**	PEL027/M	✓ (*1/*2)	✓ (*1/*2)	✓ (*1/*2 or *34/*39)	*1/*2	
**HG01981**	PEL027/C	✓ (*1/*2)	✓ (*1/*2)	✓ (*1/*2 or *34/*39)	*1/*2	
**HG02259**	PEL042/F	✓ (*1/*2)	✓ (*1/*2)	✓ (*1/*2 or *34/*39)	*1/*2	
**HG02260**	PEL042/M	✓ (*1/*1)	✓ (*1/*1)	✓ (*1/*1)	*1/*1	
**HG02261**	PEL042/C	✓ (*1/*2)	✓ (*1/*2)	✓ (*1/*2 or *34/*39)	*1/*2	
**NA06984**	1328/F	✓ (*4/*68+*4)	✗ (*4/*4)	✗ (*4/*4)	*4/*68+*4	Fusion (*68)
**NA06989**	1328/M	✓ (*9/*9)	✓ (*9/*9)	✓ (*9/*9)	*9/*9	
**NA12331**	1328/C	✓ (*4/*9)	✓ (*4/*9)	✓ (*4/*9)	*4/*9	
**NA07357**	1345/F	✓ (*1/*6)	✓ (*1/*6)	✓ (*1/*6)	*1/*6	
**NA07345**	1345/M	✓ (*1/*1)	✓ (*1/*1)	✓ (*1/*1)	*1/*1	
**NA07348**	1345/C	✓ (*1/*6)	✓ (*1/*6)	✓ (*1/*6)	*1/*6	
**NA10853**	1349/F	✓ (*2/*41)	✓ (*2/*41)	✗ (*2/*2)	*2/*41	
**NA10854**	1349/M	✓ (*1/*4)	✓ (*1/*4)	✓ (*1/*4)	*1/*4	
**NA11834**	1349/C	✓ (*2/*4)	✓ (*2/*4)	✗ (*4/*65)	*2/*4	
**NA10860**	1362/F	✓ (*1/*4+*4)	✗ (*1/*4)	✓ (*39/*4+*4 or *1/*4+*4)	*1/*4	Duplication; Case (5)
**NA10861**	1362/M	✓ (*4/*35)	✓ (*4/*35)	✗ (*4/*65)	*4/*2	Case (1)
**NA11984**	1362/C	✓ (*1/*35)	✓ (*1/*35)	✓ (*1/*35)	*1/*2	Case (1)

**NA11891**	1377/F	✓ (*1/*1)	✓ (*1/*1)	✓ (*1/*1)	*1/*1	
**NA11892**	1377/M	✓ (*6/*41)	✓ (*6/*41)	✗ (*2/*6)	*6/*41	
**NA10865**	1377/C	✓ (*1/*41)	✓ (*1/*41)	✗ (*1/*2)	*1/*41	
**NA12003**	1420/F	✓ (*4/*35)	✓ (*4/*35)	✗ (*4/*65)	*4/*2 or *4/*35	Case (1)
**NA12004**	1420/M	✓ (*2/*41)	✓ (*2/*41)	✗ (*2/*2)	*2/*41	
**NA10838**	1420/C	✓ (*2/*4)	✓ (*2/*4)	✗ (*4/*65)	*2/*4	
**NA12155**	1408/F	✓ (*1/*5)	✗ (*1/*1)	✓ (*5/*1)	*1/*5	Deletion (*5)
**NA12156**	1408/M	✓ (*1/*4)	✓ (*1/*4)	✓ (*1/*4)	*1/*4	
**NA10831**	1408/C	✓ (*4/*5)	✗ (*4/*4)	✓ (*5/*4)	*4/*5	Deletion (*5)
**NA12272**	1418/F	✓ (*1/*1)	✓ (*1/*1)	✓ (*1/*1)	*1/*1	
**NA12273**	1418/M	✓ (*1/*1)	✓ (*1/*1)	✓ (*1/*1)	*1/*1	
**NA10837**	1418/C	✓ (*1/*1)	✓ (*1/*1)	✓ (*1/*1)	*1/*1	
**NA12342**	1330/F	✓ (*4/*41)	✓ (*4/*41)	✗ (*4/*65)	*4/*41	
**NA12343**	1330/M	✓ (*1/*5)	✗ (*1/*1)	✓ (*5/*1)	*1/*5	Deletion (*5)
**NA12336**	1330/C	✓ (*5/*41)	✗ (*41/*41)	✗ (*5/*2)	*5/*41	Deletion (*5)
**NA12399**	1354/F	✓ (*1/*1)	✓ (*1/*1)	✓ (*1/*1)	*1/*1	
**NA12400**	1354/M	✓ (*1/*68+*4)	✗ (*1/*4)	✗ (*1/*4)	*1/*68+*4	Fusion (*68)
**NA12386**	1354/C	✓ (*1/*1)	✓ (*1/*1)	✓ (*1/*1)	*1/*1	
**NA12750**	1444/F	✓ (*2/*2)	✓ (*2/*2)	✓ (*2/*2)	*2/*2	
**NA12751**	1444/M	✓ (*1/*2)	✓ (*1/*2)	✓ (*1/*2 or *34/*39)	*1/*2	
**NA12740**	1444/C	✓ (*1/*2)	✓ (*1/*2)	✓ (*1/*2 or *34/*39)	*1/*2	
**NA12801**	1454/F	✓ (*4/*6)	✓ (*4/*6)	✓ (*4/*6)	*4/*6	
**NA12802**	1454/M	✓ (*2/*41)	✓ (*2/*41)	✗ (*2/*2)	*2/*41	
**NA12805**	1454/C	✓ (*2/*4)	✓ (*2/*4)	✗ (*65/*65)	*2/*4	
**NA12891**	1463/F	✓ (*41/*68+*4)	✗ (*4/*41)	✗ (*4/*65)	*41/*68+*4	Fusion (*68)
**NA12892**	1463/M	✓ (*2/*3)	✓ (*2/*3)	✓ (*2/*3)	*2/*3	
**NA12878**	1463/C	✓ (*3/*68+*4)	✗ (*3/*4)	✗ (*3/*4)	*3/68+*4	Fusion (*68)
**NA18507**	Y009/F	✓ (*2/*4+*4)	✗ (*2/*4)	✓ (*2/*4+*4)	*2/*4×*N*	Multiplication
**NA18508**	Y009/M	✓ (*2/*5)	✗ (*2/*2)	✓ (*5/*2)	*2/*5	Deletion (*5)
**NA18506**	Y009/C	✓ (*2/*5)	✗ (*2/*2)	✓ (*5/*2)	*2/*5	Deletion (*5)
**NA18516**	Y013/F	✓ (*1/*17)	✓ (*1/*17)	✓ (*1/*17)	*1/*17	
**NA18517**	Y013/M	✓ (*5/*10)	✗ (*10/*10)	✓ (*5/*10)	*5/*10	Deletion (*5)
**NA18515**	Y013/C	✓ (*1/*10)	✓ (*1/*10)	✓ (*1/*10)	*1/*10	
**NA19128**	Y077/F	✓ (*17/*17)	✓ (*17/*17)	✓ (*17/*17)	*17/*17	
**NA19127**	Y077/M	✓ (*2/*17)	✓ (*2/*17)	✓ (*2/*17)	*2/*17	
**NA19129**	Y077/C	✓ (*17/*17)	✓ (*17/*17)	✓ (*17/*17)	*17/*17	
**NA19200**	Y045/F	✓ (*1/*5)	✗ (*1/*1)	✓ (*5/*1)	(*76?)+*1/*5 or *1/*5	Deletion (*5); Case (4)

**NA19201**	Y045/M	✓ (*1/*17)	✓ (*1/*17)	✓ (*1/*17)	*1/*17	
**NA19202**	Y045/C	✓ (*1/*1)	✓ (*1/*1)	✓ (*1/*1)	(*76?)+*1/*1	Case (4)
**NA19239**	Y117/F	✓ (*15/*17)	✓ (*15/*17)	✓ (*15/*17)	*13-like?/*17 or *15/*17	Case (3)
**NA19238**	Y117/M	✓ (*1/*17)	✓ (*1/*17)	✓ (*1/*17)	*1/*17	
**NA19240**	Y117/C	✓ (*15/*17)	✓ (*15/*17)	✓ (*15/*17)	*13-like?/*17	Case (3)
**NA19685**	M011/F	✓ (*1/*2+*2)	✗ (*1/*2)	✓ (*1/*2+*2 or *2/*39+*34)	*1/*2×2	Duplication
**NA19684**	M011/M	✓ (*1/*4)	✓ (*1/*4)	✓ (*1/*4)	*1/*4	
**NA19686**	M011/C	✓ (*1/*1)	✓ (*1/*1)	✓ (*1/*1)	*1/*1	
**NA19700**	2367/F	✓ (*4/*29)	✓ (*4/*29)	✓ (*4/*29)	*4/*29	
**NA19701**	2367/M	✓ (*1/*17)	✓ (*1/*17)	✗ (*1/*2)	*1/*17	
**NA19702**	2367/C	✓ (*4/*17)	✓ (*4/*17)	✓ (*4/*17)	*4/*17	
**NA19771**	M031/F	✓ (*2/*4)	✗ (*4/*17)	✓ (*2/*4 or *4/*39)	*2/*4	
**NA19770**	M031/M	✓ (*1/*2)	✓ (*1/*2)	✓ (*1/*2 or *34/*39)	*1/*2	
**NA19772**	M031/C	✓ (*2/*4)	✓ (*2/*4)	✗ (*4/*65)	*2/*4	
**NA19789**	M037/F	✓ (*1/*1)	✓ (*1/*1)	✓ (*1/*1)	*1/*1	
**NA19788**	M037/M	✓ (*2/*78+*2)	✗ (*2/*2)	✗ (*2/*2+*2)	*2/*78+*2	Fusion (*78)
**NA19790**	M037/C	✓ (*1/*78+*2)	✗ (*1/*2)	✗ (*1/*2 or *34/*39)	*1/*78+*2	Fusion (*78)
**NA19818**	2418/F	✓ (*1/*17)	✓ (*1/*17)	✓ (*1/*17)	*1/*17	
**NA19819**	2418/M	✓ (*2/*4+*4)	✗ (*2/*4)	✗ (*65/*4+*4)	*2/*4×2	Duplication
**NA19828**	2418/C	✓ (*2/*17)	✓ (*2/*17)	✓ (*2/*17)	*2/*17	
**NA19834**	2424/F	✓ (*2/*45)	✓ (*2/*45)	✗ (*2/*2)	*2/*2	Case (2)
**NA19835**	2424/M	✓ (*1/*45)	✓ (*1/*45)	✗ (*1/*2)	*1/*2	Case (2)
**NA19836**	2424/C	✓ (*1/*45)	✓ (*1/*45)	✗ (*1/*2)	*1/*2	Case (2)
**NA19900**	2425/F	✓ (*3/*29)	✓ (*3/*29)	✓ (*3/*29)	*3/*29	
**NA19901**	2425/M	✓ (*1/*1)	✓ (*1/*1)	✓ (*1/*1)	*1/*1	
**NA19902**	2425/C	✓ (*1/*29)	✓ (*1/*29)	✓ (*1/*29)	*1/*29	

Supplementary Table 1: *CYP2D6* genotypes inferred by Aldy on the set of 32 Coriell trios (96 PGRNseq v.2 samples). In the Family column, F stands for father, M for mother, and C for child. Gene duplications, deletions and fusions are annotated in the Comments column. All calls are reported in terms of star-allele calls, since clinical pharmacogenomics and all other tools use this format for reporting genotypes. Each fusion has different number based on the fusion type and breakpoint (e.g. *78 is *CYP2D7/2D6* hybrid with the breakpoint in intron 4, while *36 denotes *10-like allele with *CYP2D7* gene conversion in exon 9). Correct predictions are marked with ✓, while incorrect predictions are marked with ✗. As can be seen, Aldy was able to successfully identify all alleles.

For few samples, validation calls were not complete, and such cases are marked with "Cases" mark in the Comments column. Those cases are as follows: cases (1) and (2) refer to allele *35 and *45 being called as *2 due to the inability of genotyping assays to properly identify those alleles; case (3) refers to the case where allele *15 is misidentified by the assays; in case (4), calls were not clear, and external validation confirmed that *76 was not present; finally, case (5) refers to the case where gene duplication was missed by validation panels; it was later confirmed that such duplication exists by the analysis of Illumina WGS samples. Further details are available in the Discussion section below the table.

3.2 Dataset (3): *CYP2D6* on Illumina WGS data

Sample	Family	Predictions			Validation	Comments
		Aldy	Astrolabe	Cypiripi		
**NA11992**	1362/F	*4/*4+*4	*4/*4	N/A	N/A	Multiplication
**NA11993**	1362/M	✓ (*1/*9)	✓ (*1/*9)	✓ (*1/*9)	*1/*9	
**NA10860**	1362/C	✓ (*1/*4+*4)	✗ (*1/*4)	✓ (*1/*4+*4)	*1/*4	Multiplication
**NA11832**	1350/M	✓ (*1/*68+*4)	✗ (*2/*4)	N/A	*1/*4	Fusion (*68); Case (1)
**NA12877**	1463/F	✓ (*4/*68+*4)	✗ (*4/*4)	✗ (Crash)	*4/*68+*4	Fusion (*68)
**NA12878**	1463/M	✓ (*3/*68+*4)	✗ (*3/*4)	✗ (*3/*4)	*3/*68+*4	Fusion (*68)
**NA12889**	1463/GF	✓ (*4/*41)	✓ (*4/*41)	✗ (*2/*4)	*4/*41	
**NA12890**	1463/GM	*68+*4/*68+*4	*4/*4	*4/*4	N/A	Fusion (*68)
**NA12891**	1463/GF	✓ (*41/*68+*4)	✗ (*4/*41)	✗ (*4/*4)	*41/*68+*4	Fusion (*68)
**NA12892**	1463/GM	✓ (*2/*3)	✓ (*2/*3)	✓ (*2/*3)	*2/*3	
**NA12879**	1463/C	*3/*68+*4	*2/*3	*3/*4	N/A	Fusion (*68)
**NA12880**	1463/C	*68+*4/*68+*4	*2/*4	*4/*4	N/A	Fusion (*68)
**NA12881**	1463/C	*68+*4/*68+*4	*2/*4	*4/*4	N/A	Fusion (*68)
**NA12882**	1463/C	✓ (*4/*68+*4)	✗ (*2/*4)	✗ (*4/*4)	*4/*68+*4	Fusion (*68)
**NA12883**	1463/C	*3/*68+*4	*2/*3	*3/*4	N/A	Fusion (*68)
**NA12884**	1463/C	*4/*68+*4	*2/*4	*4/*4	N/A	Fusion (*68)
**NA12885**	1463/C	*68+*4/*68+*4	*2/*4	*4/*4	N/A	Fusion (*68)
**NA12886**	1463/C	*3/*4	*2/*3	*3/*4	N/A	
**NA12887**	1463/C	*4/*68+*4	*2/*4	*4/*4	N/A	Fusion (*68)
**NA12888**	1463/C	*4/*68+*4	*2/*4	*4/*4	N/A	Fusion (*68)
**NA12893**	1463/C	*3/*4	*2/*3	*3/*4	N/A	
**NA19239**	Y117/F	✓ (*15/*17)	✗ (*1/*2)	✗ (*1/*17)	*15/*17	
**NA19238**	Y117/M	✓ (*1/*17)	✗ (*2/*17)	✓ (*1/*17)	*1/*17	
**NA19240**	Y117/C	✓ (*15/*17)	✗ (*1/*2)	✗ (*1/*17)	*15/*17	
**NA19900**	2425/F	✓ (*3/*29)	✗ (*17/*29)	✗ (*2/*3)	*3/*29	

Supplementary Table 2: *CYP2D6* genotypes inferred by Aldy on the set of 25 publicly available Illumina WGS samples from multiple families. Family relationships are indicated as (G)F/M: (grand)father/mother, C: child. All calls are reported in terms of star-allele calls, since clinical pharmacogenomics and all other tools use this format for reporting genotypes. Gene duplications and fusions are annotated in the Comments column. Correct predictions are marked with ✓, while incorrect predictions are marked with ✗. We had no external validations for some samples, however all predictions made by Aldy match the Mendelian laws of inheritance. For some samples, we had no access to the whole datasets which were necessary to run Cypiripi; such samples are marked with N/A. As can be seen, Aldy was able to successfully identify all alleles. The only detected fusion in these samples is denoted with *68, and case (1) refers to the case when *68 was missed by validation panels.

3.3 Dataset (2): 10 ADME genes on 137 GeT-RM (PGRNseq v.1) samples

Sample	*CYP2A6*	*CYP2C19*	*CYP2C8*	*CYP2C9*	*CYP2D6*	*CYP3A4*	*CYP3A5*	*CYP4F2*	*DPYD*	*TPMT*
✓ Matches	110	128	137	136	107	131	137	121	79	135
✓ Improvements	18	9	0	1	27	6	0	16	56	2
✗ Mismatches	3	0	0	0	0	0	0	0	0	0
? Unknown	6	0	0	0	3	0	0	0	2	0
HG00276	✓ *1/*1	✓ *1/*1	✓ *1/*3	✓ *1/*2	✓ *4/*5	✓ *1/*2	✓ *3/*3	✓ *1/*1	✓ *1/*1	✓ *1/*16
HG00436	✗ *4/*4	✓ *1/*1	✓ *1/*1	✓ *1/*1	✓ *71/*2+*2	✓ *1/*1	✓ *3/*3	✓ *1/*4 or *2/*3	✓ *1/*1	✓ *1/*1
HG00589	✓ *1/*1	✓ *1/*1	✓ *1/*1	✓ *1/*1	✓ *1/*21	✓ *1/*1	✓ *3/*3	✓ *1/*1	✓ *1/*5	✓ *1/*3
HG01190	✓ *1/*1	✓ *1/*2	✓ *1/*3	✓ *1/*2	✓ *4/*68	✓ *1/*1	✓ *1/*1	✓ *1/*3	✓ *1/*9	✓ *1/*1
NA06991	✓ *1/*1	✓ *1/*1	✓ *1/*1	✓ *1/*1	✓ *1/*4	✓ *1/*1	✓ *3/*3	✓ *1/*1	✓ *1/*4	✓ *1/*1
NA06993	✓ *1/*1	✓ *1/*1	✓ *1/*3	✓ *1/*2	✓ *4/*35	✓ *1/*22	✓ *3/*3	✓ *1/*1	✓ *4/*5	✓ *1/*1
NA07000	✓ *1/*1	✓ *1/*17	✓ *1/*1	✓ *1/*1	✓ *9/*35	✓ *1/*1	✓ *1/*3	✓ *3/*4	✓ *1/*1	✓ *1/*1
NA07019	✓ *1/*1	✓ *1/*17	✓ *1/*1	✓ *1/*1	✓ *1/*4	✓ *1/*1	✓ *3/*3	✓ *1/*3	✓ *1/*1	✓ *1/*1
NA07029	✓ *1/*1	✓ *8/*17	✓ *1/*3	✓ *1/*2	✓ *1/*35	✓ *1/*1	✓ *1/*3	✓ *3/*4	✓ *1/*1	✓ *1/*1
NA07048	? *1/*21	✓ *1/*17	✓ *1/*1	✓ *1/*1	✓ *1/*4	✓ *1/*1	✓ *3/*3	✓ *1/*1	✓ *1/*1	✓ *1/*1
NA07055	✓ *1/*1	✓ *1/*17	✓ *1/*1	✓ *1/*1	✓ *4/*4	✓ *1/*1	✓ *3/*3	✓ *1/*1	✓ *1/*5	✓ *1/*1
NA07056	✓ *1/*1	✓ *1/*1	✓ *1/*1	✓ *1/*1	✓ *2/*4	✓ *1/*22	✓ *3/*3	✓ *3/*4	✓ *1/*1	✓ *1/*1
NA07348	✓ *1/*1	✓ *2/*17	✓ *1/*4	✓ *1/*1	✓ *1/*6	✓ *1/*1	✓ *3/*3	✓ *1/*1	✓ *6/*9	✓ *1/*1
NA07357	✓ *1/*1	✓ *2/*17	✓ *1/*4	✓ *1/*1	✓ *1/*6	✓ *1/*1	✓ *3/*3	✓ *1/*1	✓ *5/*6 ★	✓ *1/*1
NA07439	✓ *1/*1	✓ *2/*27 ★	✓ *1/*1	✓ *1/*9	✓ *41/*4+*4	✓ *1/*1	✓ *1/*1	✓ *1/*2	✓ *1/*1	✓ *1/*1
NA10831	✓ *1/*2	✓ *1/*17	✓ *1/*1	✓ *1/*2	✓ *4/*5	✓ *1/*1	✓ *3/*3	✓ *1/*1	✓ *5/*9	✓ *1/*1
NA10838	✓ *1/*1	✓ *1/*1	✓ *1/*1	✓ *1/*1	✓ *2/*4	✓ *1/*1	✓ *3/*3	✓ *1/*1	✓ *1/*1	✓ *1/*1
NA10846	✓ *1/*1	✓ *1/*17	✓ *1/*1	✓ *1/*1	✓ *1/*4	✓ *1/*1	✓ *3/*3	✓ *1/*3	✓ *1/*9	✓ *1/*1
NA10847	✓ *1/*1	✓ *1/*1	✓ *1/*1	✓ *1/*1	✓ *1/*41	✓ *1/*1	✓ *3/*3	✓ *1/*1	✓ *5/*9	✓ *1/*1
NA10851	✓ *1/*2	✓ *1/*17	✓ *1/*1	✓ *1/*1	✓ *1/*4	✓ *1/*1	✓ *3/*3	✓ *1/*1	✓ *1/*9	✓ *1/*1
NA10854	✓ *1/*1	✓ *1/*1	✓ *3/*3	✓ *2/*2	✓ *1/*4	✓ *1/*1	✓ *1/*3	✓ *1/*3	✓ *1/*1	✓ *1/*1
NA10855	✓ *1/*2	✓ *1/*1	✓ *1/*3	✓ *2/*3	✓ *1/*68+*4	✓ *1/*1	✓ *3/*3	✓ *1/*1	✓ *1/*5	✓ *1/*32
NA10856	✓ *1/*1	✓ *1/*2	✓ *1/*1	✓ *1/*2	✓ *1/*5	✓ *1/*1	✓ *1/*3	✓ *1/*1	✓ *5/*6	✓ *1/*1
NA10859	✓ *1/*2	✓ *1/*1	✓ *1/*1	✓ *1/*1	✓ *1/*2	✓ *1/*1	✓ *3/*3	✓ *1/*4 or *2/*3	✓ *5/*6 ★	✓ *1/*1
NA10865	✓ *1/*9	✓ *8/*17	✓ *1/*3	✓ *1/*2	✓ *1/*41	✓ *1/*1	✓ *3/*3	✓ *1/*1	✓ *1/*1	✓ *1/*1
NA11832	✓ *1/*2	✓ *1/*2	✓ *1/*1	✓ *1/*3	✓ *1/*68+*4	✓ *1/*1	✓ *3/*3	✓ *1/*1	✓ *1/*5	✓ *1/*1
NA11839	✓ *1/*9	✓ *1/*1	✓ *1/*3	✓ *2/*3	✓ *1/*2	✓ *1/*1	✓ *1/*3	✓ *1/*3	✓ *1/*5	✓ *1/*1
NA11881	✓ *1/*1	✓ *1/*17	✓ *1/*1	✓ *1/*1	✓ *2/*3	✓ *1/*1	✓ *3/*3	✓ *1/*4 or *2/*3	✓ *1/*5	✓ *1/*1
NA11993	✓ *9/*17	✓ *1/*1	✓ *1/*1	✓ *1/*1	✓ *1/*9	✓ *1/*1	✓ *3/*3	✓ *1/*3	✓ *1/*5	✓ *1/*1
NA12003	✓ *1/*1	✓ *1/*1	✓ *1/*3	✓ *1/*2	✓ *4/*35	✓ *1/*1	✓ *1/*3	✓ *1/*1	✓ *1/*1	✓ *1/*1
NA12006	✓ *1/*1	✓ *1/*1	✓ *1/*1	✓ *1/*1	✓ *4/*41	✓ *1/*3	✓ *3/*3	✓ *1/*3	✓ *1/*1	✓ *1/*1
NA12145	✓ *1/*1	✓ *2/*17	✓ *1/*4	✓ *1/*1	✓ *1/*4	✓ *1/*1	✓ *3/*3	✓ *1/*1	✓ *1/*9	✓ *1/*1
NA12156	✓ *1/*1	✓ *1/*1	✓ *1/*1	✓ *1/*2	✓ *1/*4	✓ *1/*1	✓ *3/*3	✓ *1/*4 or *2/*3	✓ *1/*5	✓ *1/*1
NA12236	✓ *1/*1	✓ *1/*17	✓ *1/*1	✓ *1/*1	✓ *1/*4	✓ *1/*1	✓ *3/*3	✓ *1/*1	✓ *9/*9	✓ *1/*1
NA12336	? *1+*1/*2	✓ *17/*17	✓ *1/*1	✓ *1/*1	✓ *5/*41	✓ *1/*1	✓ *3/*3	✓ *1/*1	✓ *1/*9	✓ *1/*1
NA12375	✓ *1/*1	✓ *2/*17	✓ *1/*1	✓ *1/*1	✓ *1/*1	✓ *1/*1	✓ *3/*3	✓ *1/*1	✓ *1/*1	✓ *1/*1
NA12717	✓ *1/*1	✓ *2/*2	✓ *1/*1	✓ *1/*1	✓ *1/*1	✓ *1/*22	✓ *1/*3	✓ *1/*3	✓ *1/*1	✓ *1/*1

NA12753	✓ *1/*1	✓ *2/*27	✓ *1/*4	✓ *1/*9	✓ *2/*3	✓ *1/*1	✓ *3/*3	✓ *1/*1	✓ *1/*9	✓ *1/*3
NA12813	✓ *1/*1	✓ *1/*17	✓ *1/*1	✓ *1/*3	✓ *2/*4	✓ *1/*1	✓ *3/*3	✓ *1/*3	✓ *1/*4	✓ *1/*1
NA12815	✗ *1/*12	✓ *1/*2	✓ *1/*1	✓ *1/*8	✓ *2/*41	✓ *1/*1	✓ *3/*3	✓ *1/*3	✓ *1/*9	✓ *1/*1
NA12873	✓ *1/*9	✓ *1/*17	✓ *1/*1	✓ *1/*1	✓ *1/*5	✓ *1/*1	✓ *3/*3	✓ *1/*1	✓ *1/*1	✓ *1/*1
NA12878	✓ *1/*1	✓ *1/*2	✓ *1/*3	✓ *1/*2	✓ *3/*68+*4	✓ *1/*1	✓ *3/*3	✓ *1/*1	✓ *4/*5	✓ *1/*1
NA12892	✓ *1/*1	✓ *1/*1	✓ *1/*3	✓ *1/*2	✓ *2/*3	✓ *1/*22	✓ *3/*3	✓ *1/*1	✓ *1/*5	✓ *1/*1
NA15245	✓ *1/*1	✓ *1/*2	✓ *1/*4	✓ *10/*12	✓ *4/*4+*4	✓ *1/*14	✓ *3/*3	✓ *1/*1	✓ *1/*5	✓ *1/*3
NA17012	✓ *1/*1	✓ *1/*2	✓ *1/*1	✓ *1/*1	✓ *5/*10 ★	✓ *1/*1	✓ *3/*3	✓ *1/*1	✓ *1/*5	✓ *1/*1
NA17061	✓ *17/*17	✓ *2/*2	✓ *1/*1	✓ *1/*1	✓ *1/*2	✓ *1/*1	✓ *3/*3	✓ *1/*4 or *2/*3	✓ *1/*9	✓ *1/*3
NA17074	✓ *1/*1	✓ *12/*17	✓ *2/*2	✓ *1/*1	✓ *1/*2	✓ *1/*1	✓ *3/*3	✓ *1/*4 or *2/*3	✓ *1/*5	✓ *1/*1
NA17102	✓ *1/*1	✓ *1/*17	✓ *1/*1	✓ *5/*36	✓ *1/*40	✓ *1/*1	✓ *3/*3	✓ *1/*4 or *2/*3	✓ *5/*9	✓ *1/*1
NA17204	✓ *1/*1	✓ *1/*1	✓ *1/*1	✓ *1/*3	✓ *1/*35	✓ *1/*1	✓ *3/*3	✓ *1/*1	? *5/*9	✓ *1/*1
NA17227	✓ *9/*14	✓ *1/*1	✓ *1/*1	✓ *1/*2	✓ *1/*9	✓ *1/*1	✓ *3/*3	✓ *1/*4 or *2/*3	✓ *1/*1	✓ *1/*1
NA17234	✓ *1/*9	✓ *1/*1	✓ *1/*1	✓ *1/*3	✓ *1/*41	✓ *1/*1	✓ *3/*3	✓ *1/*1	✓ *1/*6	✓ *1/*1
NA17235	✓ *1/*1	✓ *1/*1	✓ *1/*1	✓ *1/*1	✓ *1/*5	✓ *1/*1	✓ *3/*3	✓ *1/*1	✓ *1/*5	✓ *1/*1
NA17244	✓ *1/*1	✓ *1/*1	✓ *1/*1	✓ *1/*1	? *2+*2/*4+*4	✓ *1/*1	✓ *3/*3	✓ *1/*1	✓ *1/*9	✓ *1/*1
NA17288	✓ *1/*1	✓ *2/*17	✓ *1/*4	✓ *1/*1	✓ *1/*1	✓ *1/*1	✓ *3/*3	✓ *1/*3	✓ *5/*9	✓ *1/*1
NA17290	✓ *1/*1	✓ *1/*2	✓ *1/*1	✓ *1/*3	✓ *1/*41	✓ *1/*1	✓ *3/*3	✓ *3/*3	✓ *1/*5	✓ *1/*1
NA17448	✓ *1/*1	✓ *1/*13	✓ *1/*2	✓ *1/*1	✓ *1/*28	✓ *1/*1	✓ *3/*3	✓ *3/*3	✓ *5/*9	✓ *1/*1
NA17454	✓ *1/*9	✓ *1/*1	✓ *1/*1	✓ *1/*8	? *2/*1+*1	✓ *1/*1	✓ *1/*3	✓ *1/*3	✓ *1/*5	✓ *1/*1
NA17641	✓ *1/*1	✓ *2/*17	✓ *1/*4	✓ *1/*1	✓ *2/*35	✓ *1/*1	✓ *3/*3	✓ *1/*1	✓ *1/*5	✓ *1/*3
NA17642	✓ *1/*1	✓ *1/*1	✓ *1/*1	✓ *1/*3	✓ *1/*1	✓ *1/*1	✓ *3/*3	✓ *1/*1	✓ *5/*9	✓ *1/*1
NA17657	✓ *1/*1	✓ *1/*1	✓ *1/*1	✓ *1/*1	✓ *4/*9	✓ *1/*1	✓ *1/*3	✓ *1/*4 or *2/*3	✓ *1/*5	✓ *1/*1
NA17658	? *1/*35	✓ *1/*17	✓ *1/*1	✓ *1/*1	✓ *1/*2	✓ *1/*1	✓ *3/*3	✓ *1/*4 or *2/*3	✓ *1/*9	✓ *1/*1
NA17660	✓ *1/*9	✓ *1/*1	✓ *1/*1	✓ *1/*1	✓ *1/*2	✓ *1/*1	✓ *3/*3	✓ *1/*1	✓ *5/*9	✓ *1/*1
NA17673	✓ *1/*9	✓ *1/*2	✓ *1/*1	✓ *1/*1	✓ *1/*4	✓ *1/*1	✓ *3/*3	✓ *1/*1	✓ *4/*6	✓ *1/*3
NA17679	✓ *9/*14	✓ *2/*17	✓ *1/*1	✓ *1/*1	✓ *1/*4	✓ *1/*1	✓ *3/*3	✓ *1/*4 or *2/*3	✓ *1/*9	✓ *1/*1
NA17702	✓ *1/*9	✓ *1/*1	✓ *1/*3	✓ *1/*2	✓ *1/*35	✓ *1/*1	✓ *3/*3	✓ *1/*1	✓ *1/*9	✓ *1/*3
NA18484	✓ *1/*9	✓ *2/*27	✓ *1/*4	✓ *1/*9	✓ *1/*17	✓ *1/*1	✓ *1/*7	✓ *1/*2	✓ *1/*9	✓ *1/*1
NA18509	✓ *1/*17	✓ *2/*2	✓ *1/*1	✓ *1/*1	✓ *2/*17	✓ *1/*1	✓ *1/*7	✓ *1/*4 or *2/*3	? *5/*9	✓ *1/*1
NA18518	✓ *1/*17	✓ *2/*17	✓ *1/*2	✓ *1/*1	✓ *17/*29	✓ *1/*1	✓ *1/*6	✓ *1/*2	✓ *5/*9	✓ *1/*1
NA18519	✓ *1/*1	✓ *1/*17	✓ *1/*2	✓ *1/*5	✓ *1/*29	✓ *1/*1	✓ *1/*6	✓ *1/*4 or *2/*3	✓ *9/*9	✓ *1/*1
NA18524	✓ *1/*1	✓ *1/*2	✓ *1/*1	✓ *1/*3	✓ *1/*36+*10	✓ *1/*1	✓ *1/*3	✓ *1/*3	✓ *1/*5	✓ *1/*1
NA18526	✓ *1/*9	✓ *1/*1	✓ *1/*1	✓ *1/*1	✓ *1/*36+*10+*36	✓ *1/*1	✓ *1/*1	✓ *1/*3	✓ *1/*1	✓ *1/*1
NA18540	✓ *1/*1	✓ *1/*2	✓ *1/*1	✓ *1/*1	✓ *36/*36+*10+*41	✓ *1/*1	✓ *1/*3	✓ *1/*3	✓ *1/*9	✓ *1/*1
NA18544	✗ *1/*19	✓ *1/*2	✓ *1/*1	✓ *1/*1	✓ *10/*41	✓ *1/*1	✓ *1/*3	✓ *1/*1	✓ *1/*5	✓ *1/*1
NA18552	✓ *9/*9	✓ *1/*4	✓ *1/*1	✓ *1/*1	✓ *1/*14	✓ *1/*1	✓ *3/*3	✓ *1/*3	✓ *1/*5	✓ *1/*1
NA18563	✓ *1/*1	✓ *1/*1	✓ *1/*1	✓ *1/*3	✓ *1/*36+*10	✓ *1/*1	✓ *1/*1	✓ *1/*1	✓ *1/*5	✓ *1/*1
NA18564	✓ *1/*1	✓ *2/*3	✓ *1/*1	✓ *1/*1	✓ *2/*36+*10	✓ *1/*1	✓ *1/*1	✓ *3/*4	✓ *5/*9	✓ *1/*1
NA18565	✓ *4/*15	✓ *1/*1	✓ *1/*1	✓ *1/*1	✓ *10/*36+*10	✓ *1/*1	✓ *1/*3	✓ *1/*1	✓ *1/*1	✓ *1/*1
NA18572	✓ *1/*1	✓ *1/*1	✓ *1/*1	✓ *1/*1	✓ *41/*36+*10	✓ *1/*1	✓ *1/*3	✓ *1/*3	✓ *1/*5	✓ *1/*1
NA18617	✓ *1/*4	✓ *1/*2	✓ *1/*1	✓ *1/*1	✓ *36+*10/*36+*10	✓ *1/*1	✓ *3/*3	✓ *1/*1	✓ *1/*1	✓ *1/*1
NA18855	✓ *1/*1	✓ *2/*27	✓ *1/*1	✓ *1/*9	✓ *1/*5	✓ *1/*1	✓ *3/*6	✓ *1/*1	✓ *9/*9	✓ *1/*3
NA18861	? *1+*1/*25	✓ *1/*1	✓ *1/*1	✓ *1/*1	✓ *5/*29	✓ *1/*1	✓ *1/*1	✓ *1/*1	✓ *1/*9	✓ *1/*1
NA18868	✓ *1/*17	✓ *1/*2	✓ *1/*1	✓ *1/*1	✓ *2/*5	✓ *1/*1	✓ *1/*3	✓ *1/*1	✓ *1/*9	✓ *1/*1

NA18873	✓ *1/*9	✓ *1/*2	✓ *1/*1	✓ *1/*8	✓ *5/*17	✓ *1/*1	✓ *1/*1	✓ *1/*1	✓ *5/*9	✓ *1/*1
NA18942	✓ *1/*4	✓ *1/*1	✓ *1/*1	✓ *1/*1	✓ *2/*2	✓ *1/*1	✓ *3/*3	✓ *1/*1	✓ *1/*1	✓ *1/*1
NA18945	✓ *4/*9	✓ *1/*2	✓ *1/*1	✓ *1/*1	✓ *1/*5	✓ *1/*1	✓ *1/*3	✓ *1/*1	✓ *1/*1	✓ *1/*1
NA18952	✓ *4/*4	✓ *1/*1	✓ *1/*1	✓ *1/*1	✓ *2/*2	✓ *1/*1	✓ *3/*3	✓ *1/*1	✓ *5/*5	✓ *1/*1
NA18959	✓ *1/*4	✓ *1/*1	✓ *1/*1	✓ *1/*3	✓ *2/*36+*10	✓ *1/*1	✓ *1/*3	✓ *1/*1	✓ *1/*1	✓ *1/*1
NA18966	✓ *1/*4	✓ *1/*1	✓ *1/*1	✓ *1/*1	✓ *1/*2	✓ *1/*16	✓ *1/*3	✓ *1/*3	✓ *1/*1	✓ *1/*3
NA18973	✓ *4/*4	✓ *1/*1	✓ *1/*1	✓ *1/*1	✓ *1/*21	✓ *1/*1	✓ *1/*3	✓ *1/*1	✓ *5/*9	✓ *1/*1
NA18980	✓ *9/*9	✓ *1/*1	✓ *1/*1	✓ *1/*1	✓ *2/*36+*10	✓ *1/*1	✓ *1/*3	✓ *1/*1	✓ *1/*5	✓ *1/*1
NA18992	✓ *9/*18	✓ *1/*1	✓ *1/*1	✓ *1/*1	✓ *1/*5	✓ *1/*1	✓ *3/*3	✓ *1/*1	✓ *1/*1	✓ *1/*1
NA19003	✓ *1/*1	✓ *1/*2	✓ *1/*1	✓ *1/*1	✓ *1/*1	✓ *1/*1	✓ *3/*3	✓ *1/*1	✓ *1/*1	✓ *1/*1
NA19007	✓ *1/*4	✓ *1/*1	✓ *1/*1	✓ *1/*1	✓ *1/*1	✓ *1/*1	✓ *3/*3	✓ *1/*3	✓ *1/*1	✓ *1/*1
NA19035	✓ *9/*17	✓ *17/*17	✓ *2/*2	✓ *1/*1	✓ *2/*5	✓ *1/*12	✓ *1/*7	✓ *1/*3	✓ *1/*1	✓ *3/*3
NA19095	✓ *9/*9	✓ *1/*1	✓ *1/*2	✓ *1/*1	✓ *1/*29	✓ *1/*1	✓ *1/*3	✓ *1/*3	✓ *9/*9	✓ *1/*1
NA19109	✓ *17/*20	✓ *17/*17	✓ *2/*2	✓ *1/*1	✓ *29/*2+*2	✓ *1/*15	✓ *1/*3	✓ *1/*1	✓ *1/*9	✓ *1/*1
NA19122	✓ *1/*35	✓ *2/*15	✓ *1/*1	✓ *1/*11	✓ *2/*17	✓ *1/*1	✓ *1/*1	✓ *1/*2	✓ *1/*1	✓ *1/*1
NA19143	✓ *1/*35	✓ *1/*15	✓ *1/*1	✓ *1/*6	✓ *10/*45	✓ *1/*1	✓ *6/*7	✓ *1/*1	✓ *1/*1	✓ *1/*1
NA19147	✓ *9/*23	✓ *1/*17	✓ *1/*1	✓ *1/*1	✓ *17/*29	✓ *1/*1	✓ *1/*3	✓ *1/*1	✓ *1/*9	✓ *1/*1
NA19174	✓ *9/*24	✓ *1/*2	✓ *1/*1	✓ *1/*1	✓ *4/*40	✓ *1/*1	✓ *1/*6	✓ *1/*1	✓ *1/*1	✓ *1/*1
NA19176	✓ *1/*1	✓ *2/*17	✓ *2/*2	✓ *1/*1	✓ *1/*2	✓ *1/*1	✓ *1/*3	✓ *1/*1	✓ *1/*1	✓ *1/*8
NA19178	✓ *1/*20	✓ *6/*27	✓ *1/*1	✓ *5/*9	✓ *1/*1	✓ *1/*1	✓ *1/*1	✓ *1/*1	✓ *1/*9	✓ *1/*1
NA19207	✓ *1/*17	✓ *2/*17	✓ *1/*2	✓ *1/*1	✓ *10/*2+*2	✓ *1/*1	✓ *3/*7	✓ *1/*1	✓ *1/*9	✓ *1/*1
NA19213	✓ *17/*24	✓ *1/*15	✓ *1/*1	✓ *1/*6	✓ *1/*1	✓ *1/*1	✓ *1/*6	✓ *1/*1	✓ *1/*1	✓ *1/*1
NA19226	✓ *1/*17	✓ *1/*2	✓ *1/*1	✓ *1/*8	✓ *2/*2+*2	✓ *1/*15	✓ *1/*6	✓ *1/*2	✓ *1/*9	✓ *1/*1
NA19238	✓ *1/*9	✓ *1/*1	✓ *1/*1	✓ *1/*1	✓ *1/*17	✓ *1/*1	✓ *1/*1	✓ *1/*2	✓ *9/*9	✓ *1/*1
NA19239	✓ *1/*17	✓ *13/*17	✓ *1/*2	✓ *1/*1	✓ *15/*17	✓ *1/*1	✓ *1/*1	✓ *1/*1	✓ *1/*9	✓ *1/*1
NA19444	✓ *17/*17	✓ *1/*17	✓ *1/*2	✓ *1/*1	✓ *1/*1	✓ *1/*1	✓ *3/*7	✓ *1/*2	✓ *1/*5	✓ *1/*1
NA19700	✓ *17/*35	✓ *1/*27	✓ *1/*1	✓ *1/*9	✓ *4/*29	✓ *1/*1	✓ *1/*3	✓ *1/*2	✓ *9/*9	✓ *1/*1
NA19785	✓ *1/*1	✓ *1/*1	✓ *1/*1	✓ *1/*1	✓ *2/*1+*79	✓ *1/*1	✓ *1/*3	✓ *3/*3	✓ *1/*1	✓ *1/*1
NA19789	✓ *1/*1	✓ *1/*1	✓ *1/*3	✓ *1/*2	✓ *1/*1	✓ *1/*1	✓ *3/*3	✓ *1/*4 or *2/*3	✓ *1/*5	✓ *1/*1
NA19819	✓ *1/*1	✓ *1/*17	✓ *1/*2	✓ *1/*1	✓ *2/*4+*4	✓ *1/*1	✓ *3/*6	✓ *1/*4 or *2/*3	✓ *5/*9	✓ *1/*1
NA19908	✓ *1/*17	✓ *1/*17	✓ *1/*1	✓ *1/*5	✓ *1/*46 or *43/*45	✓ *1/*15	✓ *1/*3	✓ *1/*4 or *2/*3	✓ *1/*9	✓ *1/*1
NA19917	✓ *1/*1	✓ *2/*15	✓ *1/*1	✓ *1/*1	✓ *1/*40	✓ *1/*1	✓ *1/*7	✓ *1/*2	✓ *9/*9	✓ *1/*1
NA19920	✓ *1/*35	✓ *1/*1	✓ *1/*1	✓ *1/*1	✓ *1/*4+*4	✓ *1/*1	✓ *7/*7	✓ *1/*1	✓ *9/*9	✓ *1/*3
NA20296	✓ *1/*1	✓ *1/*1	✓ *1/*1	✓ *1/*1	✓ *1/*2	✓ *1/*1	✓ *1/*6	✓ *1/*1	✓ *1/*9	✓ *1/*3
NA20509	✓ *1/*1	✓ *2/*2	✓ *1/*4	✓ *1/*1	✓ *4/*35	✓ *1/*1	✓ *3/*3	✓ *1/*1	✓ *1/*9	✓ *1/*1
NA21781	✓ *1/*21	✓ *1/*2	✓ *1/*1	✓ *1/*1	✓ *68+*4/*2+*2	✓ *1/*1	✓ *3/*3	✓ *1/*1	✓ *1/*5	✓ *1/*1
NA23090	✓ *1/*9	✓ *2/*2	✓ *1/*1	✓ *1/*1	✓ *1/*36+*10	✓ *1/*1	✓ *1/*3	✓ *1/*1	✓ *1/*1	✓ *1/*1
NA23093	✓ *1/*1	✓ *2/*2	✓ *1/*1	✓ *1/*1	✓ *1/*36+*10	✓ *1/*1	✓ *1/*3	✓ *3/*3	✓ *1/*1	✓ *1/*1
NA23246	✓ *1/*1	✓ *3/*17	✓ *1/*1	✓ *1/*1	✓ *36+*10/*10+*10	✓ *1/*1	✓ *3/*3	✓ *1/*3	✓ *1/*5	✓ *1/*1
NA23275	✓ *1/*1	✓ *1/*1	✓ *1/*1	✓ *5/*5	✓ *1/*40	✓ *1/*1	✓ *1/*1	✓ *1/*1	✓ *1/*5	✓ *1/*8
NA23296	✓ *1/*14	✓ *1/*1	✓ *1/*1	✓ *1/*1	✓ *4/*2+*2	✓ *1/*1	✓ *3/*3	✓ *1/*3	✓ *5/*5	✓ *1/*1
NA23297	✓ *1/*1	✓ *1/*17	✓ *1/*2	✓ *1/*1	✓ *17/*10+*10	✓ *1/*1	✓ *1/*7	✓ *1/*1	✓ *1/*9	✓ *1/*1
NA23313	✓ *1/*1	✓ *1/*17	✓ *1/*1	✓ *1/*1	✓ *2/*2+*2	✓ *1/*22	✓ *3/*3	✓ *1/*1	✓ *1/*1	✓ *1/*1
NA23348	✓ *1/*1	✓ *8/*17	✓ *1/*3	✓ *1/*2	✓ *7/*35	✓ *1/*1	✓ *3/*3	✓ *1/*4 or *2/*3	✓ *1/*5	✓ *1/*1
NA23405	✓ *1/*1	✓ *1/*2	✓ *1/*1	✓ *1/*3	✓ *1/*7	✓ *1/*1	✓ *1/*3	✓ *1/*3	✓ *1/*1	✓ *1/*1

NA23872	✓ *1/*1	✓ *1/*8	✓ *1/*3	✓ *1/*2	✓ *2/*2	✓ *1/*1	✓ *3/*3	✓ *1/*4 or *2/*3	✓ *9/*9	✓ *1/*1
NA23873	? *1+*1/*1	✓ *1/*8	✓ *3/*3	✓ *2/*2	✓ *1/*68+*4	✓ *1/*1	✓ *3/*3	✓ *1/*4 or *2/*3	✓ *2/*6	✓ *1/*1
NA23874	✓ *1/*1	✓ *2/*6	✓ *1/*1	✓ *1/*1	? *4/*4	✓ *1/*1	✓ *3/*3	✓ *1/*1	✓ *1/*5	✓ *1/*1
NA23877	✓ *1/*1	✓ *1/*2	✓ *1/*4	✓ *1/*1	✓ *15/*41	✓ *1/*1	✓ *3/*3	✓ *1/*1	✓ *1/*1	✓ *1/*1
NA23878	✓ *1/*1	✓ *1/*4 or *4/*17	✓ *1/*1	✓ *1/*1	✓ *39/*4+*4 or *83/*4+*4	✓ *1/*1	✓ *3/*3	✓ *1/*3	✓ *1/*1	✓ *1/*1
NA23881	✓ *1/*1	✓ *1/*4	✓ *1/*3	✓ *1/*2	✓ *2/*41	✓ *1/*1	✓ *3/*3	✓ *1/*1	✓ *1/*1	✓ *1/*1
NA24008	✓ *1/*9	✓ *9/*17	✓ *1/*1	✓ *1/*1	✓ *1/*68+*4	✓ *22/*22	✓ *3/*3	✓ *1/*4 or *2/*3	✓ *1/*5	✓ *1/*1
NA24009	✓ *1/*1	✓ *2/*9	✓ *1/*1	✓ *1/*1	✓ *29/*29	✓ *1/*1	✓ *3/*6	✓ *1/*4 or *2/*3	✓ *1/*5	✓ *1/*8
NA24027	? *1/*35	✓ *1/*2	✓ *1/*3	✓ *1/*2	✓ *6/*2+*2	✓ *1/*1	✓ *1/*3	✓ *1/*1	✓ *4/*5 ★	✓ *1/*1
NA24217	✓ *1/*1	✓ *1/*1	✓ *1/*3	✓ *1/*2	✓ *2/*41+*41+*41	✓ *1/*1	✓ *1/*3	✓ *3/*3	✓ *4/*9	✓ *1/*1

Supplementary Table 3: Aldy genotyping results on a set of 137 PGRNseq v.1 samples. All calls are reported in terms of star-allele calls, since clinical pharmacogenomics and all other tools use this format for reporting genotypes. The results were matched with the published validations in [2]. Matches (✓ black color) indicate that Aldy's prediction match the panel validation. Improvement (✓ green color) shows improvements over panel validation. Details of these improvements are given in Discussion section below the table. Failure (✗ red color) means that Aldy failed to find the optimal genotype. Unknown mark (? blue color) is used in case when the proper genotype is not clear. Star (★) indicates the possible presence of novel alleles.

3.4 Dataset (1): 10 ADME genes on 17 PGRNseq v.2 samples

Sample	*CYP2A6*	*CYP2C19*	*CYP2C8*	*CYP2C9*	*CYP2D6*	*CYP3A4*	*CYP3A5*	*CYP4F2*	*DPYD*	*TPMT*
✓ Matches	14	16	17	17	16	17	17	15	9	17
✓ Improvements	2	1	0	0	1	0	0	2	8	0
✗ Mismatches	0	0	0	0	0	0	0	0	0	0
? Unknown	1	0	0	0	0	0	0	0	0	0
HG01190	✓ *1/*1	✓ *1/*2	✓ *1/*3	✓ *1/*2	✓ *4/*68	✓ *1/*1	✓ *1/*1	✓ *1/*3	✓ *1/*9	✓ *1/*1
NA07348	✓ *1/*1	✓ *2/*17	✓ *1/*4	✓ *1/*1	✓ *1/*6	✓ *1/*1	✓ *3/*3	✓ *1/*1	✓ *6/*9	✓ *1/*1
NA07357	✓ *1/*1	✓ *2/*17	✓ *1/*4	✓ *1/*1	✓ *1/*6	✓ *1/*1	✓ *3/*3	✓ *1/*1	✓ *5/*6 ⋆	✓ *1/*1
NA10831	✓ *1/*2	✓ *1/*17	✓ *1/*1	✓ *1/*2	✓ *4/*5	✓ *1/*1	✓ *3/*3	✓ *1/*1	✓ *5/*9	✓ *1/*1
NA10838	✓ *1/*1	✓ *1/*1	✓ *1/*1	✓ *1/*1	✓ *2/*4	✓ *1/*1	✓ *3/*3	✓ *1/*1	✓ *1/*1	✓ *1/*1
NA10854	✓ *1/*1	✓ *1/*1	✓ *3/*3	✓ *2/*2	✓ *1/*4	✓ *1/*1	✓ *1/*3	✓ *1/*3	✓ *1/*1	✓ *1/*1
NA10865	✓ *1/*9	✓ *8/*17	✓ *1/*3	✓ *1/*2	✓ *1/*41	✓ *1/*1	✓ *3/*3	✓ *1/*1	✓ *1/*1	✓ *1/*1
NA12003	✓ *1/*1	✓ *1/*1	✓ *1/*3	✓ *1/*2	✓ *4/*35	✓ *1/*1	✓ *1/*3	✓ *1/*1	✓ *1/*1	✓ *1/*1
NA12156	✓ *1/*1	✓ *1/*1	✓ *1/*1	✓ *1/*2	✓ *1/*4	✓ *1/*1	✓ *3/*3	✓ *1/*4 or *2/*3	✓ *1/*5	✓ *1/*1
NA12336	? *1+*1/*2	✓ *17/*17	✓ *1/*1	✓ *1/*1	✓ *5/*41	✓ *1/*1	✓ *3/*3	✓ *1/*1	✓ *1/*9	✓ *1/*1
NA12878	✓ *1/*1	✓ *1/*2	✓ *1/*3	✓ *1/*2	✓ *3/*68+*4	✓ *1/*1	✓ *3/*3	✓ *1/*1	✓ *4/*5	✓ *1/*1
NA12892	✓ *1/*1	✓ *1/*1	✓ *1/*3	✓ *1/*2	✓ *2/*3	✓ *1/*22	✓ *3/*3	✓ *1/*1	✓ *1/*5	✓ *1/*1
NA19238	✓ *1/*9	✓ *1/*1	✓ *1/*1	✓ *1/*1	✓ *1/*17	✓ *1/*1	✓ *1/*1	✓ *1/*2	✓ *9/*9	✓ *1/*1
NA19239	✓ *1/*17	✓ *13/*17	✓ *1/*2	✓ *1/*1	✓ *15/*17	✓ *1/*1	✓ *1/*1	✓ *1/*1	✓ *1/*9	✓ *1/*1
NA19700	✓ *17/*35	✓ *1/*27	✓ *1/*1	✓ *1/*9	✓ *4/*29	✓ *1/*1	✓ *1/*3	✓ *1/*2	✓ *9/*9	✓ *1/*1
NA19789	✓ *1/*1	✓ *1/*1	✓ *1/*3	✓ *1/*2	✓ *1/*1	✓ *1/*1	✓ *3/*3	✓ *1/*4 or *2/*3	✓ *1/*5	✓ *1/*1
NA19819	✓ *1/*1	✓ *1/*17	✓ *1/*2	✓ *1/*1	✓ *2/*4+*4	✓ *1/*1	✓ *3/*6	✓ *1/*4 or *2/*3	✓ *5/*9	✓ *1/*1

Supplementary Table 4: Aldy genotyping results on the set of 17 PGRNseq v.2 samples. All calls are reported in terms of star-allele calls, since clinical pharmacogenomics and all other tools use this format for reporting genotypes. The results were matched with the published validations in [2]. Matches (✓ black color) indicate that Aldy's prediction match the panel validation. Improvement (✓ green color) shows improvements over panel validation. Details of these improvements are given in Discussion section below the table. Failure (✗ red color) means that Aldy failed to find the optimal genotype. Unknown mark (? blue color) is used in case when the proper genotype is not clear. Star (⋆) indicates the possible presence of novel alleles.

4 Discussion

4.1 Dataset (1): 96 PGRNseq v.2 samples

The following list explains cases where we observed the discrepency between the validation calls and Aldy's predictions. These cases are as follows:

case (1): allele *35 is called as *2 for the samples NA10861, NA11984 and NA12003. However, as reported by [2], NA12003 actually contains *35 allele instead of *2, and this discrepancy is mostly due to the inability of our assays to properly genotype *35 allele [3].

case (2): samples NA19834, NA19835 and NA19836 contain SNP c.1716 G>A. This SNP differentiates alleles *2 (called by genotyping assay) and *45 (called by Aldy and Astrolabe) and it is not present in commercially available TaqMan assays [4].

case (3): allele *15 is mis-identified. This allele is also problematic for most of the current genotyping platforms. For example, TaqMan assays often misinterpret *15 allele as some other allele [3]. One of the reasons for this is that c.137 insT, which defines the *15 allele, is also present in all *13 fusion alleles. However, [2] confirmed that NA19239's actual genotype contains *15, which matches our results as well.

case (4): copy number results for *13-like fusion allele *76 are not clear for samples NA19200 and NA19202. However, additional validation by [4] confirms that *76 is not present, which matches Aldy's prediction. Furthermore, we couldn't find any evidence of increased coverage in *CYP2D7* region, that would be suggested by the existence of *76. All those cases suggest that Aldy provides more accurate genotyping results than currently used genotyping panels, especially in the presence of recently discovered alleles.

case (5): for sample NA10860, Aldy detects a *4 allele duplication, which PCR-based methods miss. We have cross-validated our prediction by running Aldy on Illumina HiSeq X WGS NA10860 sample publicly available from `https://export.uppmax.uu.se/a2009002/opendata/HiSeqX_CEPH/`. This sample was sequenced with approx. 28× depth of coverage (or 14× per chromosome). By simple coverage analysis, it is clear that there are at least 3 copies of *CYP2D6*, since average coverage of the *CYP2D6* region is 42×. Thus, we assume that the correct allele is indeed *1/*4+*4.

4.2 Dataset (2): 137 PGRNseq v.1 samples

The following list explains the differences reported in the Supplementary Table 3, including the evidences which were used when concluding that Aldy offers improved predictions over other methods reported in [2].

CYP2A6

- No existence of *CYP2A6*8* SNP (c.6600 G>T)
  NA12003, NA18855, NA19176

- *CYP2A6*14* SNP (c.86 G>A) present
  NA17679, NA17227, NA23296
- *CYP2A6*18* SNP (c.5668 A>T) present
  NA18992
- *CYP2A6*23* SNP (c.2161 C>T) present
  NA19147
- *CYP2A6*24* SNPs present
  NA19213
- *CYP2A6*35* SNP (c.6458 A>T) present
  NA19143, NA19122, NA19920
- Deletion (*CYP2A6*4*) present
  NA18565, NA18942, NA18945
- Presence of additional SNPs in highly homologous regions recorded, but extra validation is needed:
  NA17658, NA19174, NA19700, NA07048, NA24027, NA21781, NA18544 (most likely not correct)
- Copy number calls cannot be inferred clearly because of the high level of sequencing noise:
  NA23873, NA18861, NA12336, NA12815 (most likely not correct), HG00436 (most likely not correct)

CYP2C9

All potential *CYP2C9*18* allele calls were confirmed with Aldy.

- *CYP2C9*36* SNP (c.1 A>G) present
  NA17102

CYP2C19

- *CYP2C19*12* SNPs present
  NA17074
- *CYP2C19*15* SNPs present
  NA19122, NA19917
- *CYP2C19*27* SNPs present
  NA19178, NA18484, NA18855, NA12753
- *CYP2C19*12* SNPs are not present
  NA19700
- Novel *CYP2C19* allele detected
  NA07439: novel c.19153 C>T on *CYP2C19*27* background

CYP2D6

- Extra SNPs detected:
  HG00436 (*CYP2D6*71* because of SNP c.125 G>A);
  NA19143 (*CYP2D6*45* because of SNP c.1716 G>A);
  NA17448 (*CYP2D6*28* because of SNPs c.19 G>A and c.1704 C>G);
  NA19908 (*CYP2D6*46* because of SNPs c.77 G>A and c.1716 G>A);
  NA06993, NA07000 and NA12003 (*CYP2D6*35* because of SNP c.31 G>A)

- Presence of *CYP2D6*68* fusion
  NA10855, NA11832, NA21781, NA23873, NA24008

- Precise copy number calling of each major star-allele
  NA07439 (two *CYP2D6*4* copies), NA19207 and NA23296 (two *CYP2D6*2* copies)

- Presence of *CYP2D6*79* fusion
  NA19785

- Presence of multiple copies of *CYP2D6*10*, or tandem *CYP2D6*10+CYP2D6*36* alleles
  NA18526, NA18540, NA18563, NA18572, NA18617, NA23090, NA23093, NA23246

- Detection of additional allele copies
  NA23878 (extra *CYP2D6*4*), NA24217 (extra *CYP2D6*41*)

- Novel *CYP2D6* allele discovered (c.77 G>A on *CYP2D6*10* background)
  NA17012

- Exact copy number not clear
  NA17244, NA17454 and NA23874

CYP3A4

- *CYP3A4*12* SNP (c.21896 C>T) present
  NA19035

- *CYP3A4*15* SNP (c.14269 G>A) present
  NA19109, NA19226, NA19908

- *CYP3A4*14* SNP (c.44 T>C) present
  NA15245

- *CYP3A4*16* SNP (c.15603 C>G) present
  NA18966

- *CYP3A4*12* SNP (c.21896 C>T) present
  NA19035

- *CYP3A4*15* SNP (c.14269 G>A) present
  NA19109, NA19226, NA19908

- *CYP3A4*14* SNP (c.44 T>C) present
  NA15245

- *CYP3A4*16* SNP (c.15603 C>G) present
  NA18966

CYP4F2

- *CYP4F2*2* SNP present
  NA19700, NA19444, NA19238, NA19917

- No signs of *CYP4F2*2* SNP
  NA23313, NA12006, NA18868

- All other marked samples (9 of them) had a novel *CYP4F2* allele containing SNPs from both *CYP4F2*2* and *CYP4F2*3*. We denoted this allele as *CYP4F2*4*.

TPMT

- *TPMT*16* SNPs present
  HG00276

- *TPMT*32* SNPs present
  NA10855

DPYD

- *DPYD*6* SNPs present
  NA17673, NA07348, NA23873, NA17234, NA10856

- Novel *DPYD* allele(s) discovered (c.85 A>G on *DPYD*4*, *DPYD*5* or *DPYD*6*-like background)
  NA07357, NA10859, NA24027

- Unclear genotypes (seems like combination of *DPYD*5* and *DPYD*9* alleles)
  NA17204, NA18509

- All other marked samples have either *DPYD*5* or *DPYD*9* SNPs present.

5 Reproducibility

Illumina WGS data was downloaded from:

NA12877 https://storage.googleapis.com/genomics-public-data/platinum-genomes/bam/NA12877_S1.bam
NA12878 https://storage.googleapis.com/genomics-public-data/platinum-genomes/bam/NA12878_S1.bam
NA12889 https://storage.googleapis.com/genomics-public-data/platinum-genomes/bam/NA12889_S1.bam
NA12890 https://storage.googleapis.com/genomics-public-data/platinum-genomes/bam/NA12890_S1.bam
NA12891 https://storage.googleapis.com/genomics-public-data/platinum-genomes/bam/NA12891_S1.bam
NA12892 https://storage.googleapis.com/genomics-public-data/platinum-genomes/bam/NA12892_S1.bam

NA11832 https://storage.googleapis.com/genomics-public-data/ftp-trace.ncbi.nih.gov/1000genomes/ftp/phase3/data/NA11832/alignment/NA11832.mapped.ILLUMINA.bwa.CEU.low_coverage.20120522.bam

NA19238 https://storage.googleapis.com/genomics-public-data/ftp-trace.ncbi.nih.gov/1000genomes/ftp/phase3/data/NA19238/high_coverage_alignment/NA19238.mapped.ILLUMINA.bwa.YRI.high_coverage_pcr_free.20130924.bam

NA19239 https://storage.googleapis.com/genomics-public-data/ftp-trace.ncbi.nih.gov/1000genomes/ftp/phase3/data/NA19239/high_coverage_alignment/NA19239.mapped.ILLUMINA.bwa.YRI.high_coverage_pcr_free.20130924.bam

NA19240 https://storage.googleapis.com/genomics-public-data/ftp-trace.ncbi.nih.gov/1000genomes/ftp/phase3/data/NA19240/high_coverage_alignment/NA19240.mapped.ILLUMINA.bwa.YRI.high_coverage_pcr_free.20130924.bam

NA19900 https://storage.googleapis.com/genomics-public-data/ftp-trace.ncbi.nih.gov/1000genomes/ftp/phase3/data/NA19900/alignment/NA19900.mapped.ILLUMINA.bwa.ASW.low_coverage.20120522.bam

NA10860 https://export.uppmax.uu.se/a2009002/opendata/HiSeqX_CEPH/NA10860-PCR-free-150pM/03-BAM/NA10860-PCR-free-150pM.clean.dedup.recal.bam

NA11992 https://export.uppmax.uu.se/a2009002/opendata/HiSeqX_CEPH/CEP-NA11992/03-BAM/CEP-NA11992.clean.dedup.recal.bam

NA11993 https://export.uppmax.uu.se/a2009002/opendata/HiSeqX_CEPH/NA11993-PCR-free-150pM/03-BAM/NA11993-PCR-free-150pM.clean.dedup.recal.bam

Other Platinum Genome samples were obrained from dbGaP (project phs001224).

5.1 Aldy

Aldy (version v1.0) was run as follows:

```
aldy -p profile -g gene -o aldy/sample.aldy -l aldy/sample.aldylog sample.bam
```

The profile parameter was either illumina, pgrnseq-v2 or pgrnseq-v1, depending on the platform the sample was sequenced with.

5.2 Cypiripi

Cypiripi required interleaved FASTQ files. For majority of the samples, we downloaded the matching FASTQ files and ran them with Cypiripi pipeline. However, FASTQ files for Platinum Genome samples and NA19900 were extracted from the corresponding BAM files. Sample NA11832 was not compatible with Cypiripi.

Interleaved FASTQs were processed for Cypiripi's use as follows:

```
cypiripi.py -r distribution/reference -f sample.fq
```

Cypiripi (commit 44b8949) was run as follows:

```
cypiripi -f distribution/reference.combined.align \
  -s sample.sam -C coverage -E error -T threshold \
  >cypiripi/sample.results 2>&1
```

We used following parameters for WGS samples:

Platinum Genomes: coverage is 25, threshold is 7, error is 500; except for

NA12879, NA12880, NA12884, NA12889: coverage is 20, threshold is 5, error is 500;

NA19900: coverage is 7, threshold is 2, error is 500;

NA19238, NA19239, NA19240: coverage is 14, threshold is 3, error is 500;

NA10860, NA11992, NA11993: coverage is 13, threshold is 3, error is 500.

Although Cypiripi does not support PGRNseq v.2 data (because the depth of coverage is not uniform), we managed to run Cypiripi by approximating the depth of coverage. The following values were used:

PGRNseq v.2: coverage is 350, threshold is 89, error is 10000; except for

NA19701: coverage is 330, threshold is 82, error is 10000;

HG00423, HG00464, HG01061, HG01062, HG01981, NA10861: coverage is 420, threshold is 105, error is 10000;

NA12400, NA12891, NA18507, NA19818, NA19834, NA19902: coverage is 420, threshold is 105, error is 10000;

HG00463, HG00465: coverage is 600, threshold is 150, error is 10000.

5.3 Astrolabe

Astrolabe requires VCF files for *CYP2D6* detection. For 1000 genome WGS samples (NA11832, NA19238, NA19239, NA19900 and NA19240), we extracted matching VCF files from 1000 Genome VCF files available at `ftp://ftp.1000genomes.ebi.ac.uk/vol1/ftp/release/20130502`. For other WGS samples, we used:

NA12877 `https://storage.googleapis.com/genomics-public-data/platinum-genomes/vcf/NA12877_S1.genome.vcf`

NA12878 `https://storage.googleapis.com/genomics-public-data/platinum-genomes/vcf/NA12878_S1.genome.vcf`

NA12889 `https://storage.googleapis.com/genomics-public-data/platinum-genomes/vcf/NA12889_S1.genome.vcf`

NA12890 `https://storage.googleapis.com/genomics-public-data/platinum-genomes/vcf/NA12890_S1.genome.vcf`

NA12891 `https://storage.googleapis.com/genomics-public-data/platinum-genomes/vcf/NA12891_S1.genome.vcf`

NA12892 `https://storage.googleapis.com/genomics-public-data/platinum-genomes/vcf/NA12892_S1.genome.vcf`

NA10860 `https://export.uppmax.uu.se/a2009002/opendata/HiSeqX_CEPH/NA10860-PCR-free-150pM/04-VCF/NA10860-PCR-free-150pM.clean.dedup.recal.bam.recalibrated.indel.annotated.vcf` and `https://export.uppmax.uu.se/a2009002/opendata/HiSeqX_CEPH/NA10860-PCR-free-150pM/04-VCF/NA10860-PCR-free-150pM.clean.dedup.recal.bam.recalibrated.snp.annotated.vcf`

NA11993 `https://export.uppmax.uu.se/a2009002/opendata/HiSeqX_CEPH/NA11993-PCR-free-150pM/04-VCF/NA11993-PCR-free-150pM.clean.dedup.recal.bam.recalibrated.indel.annotated.vcf` and `https://export.uppmax.uu.se/a2009002/opendata/HiSeqX_CEPH/NA11993-PCR-free-150pM/04-VCF/NA11993-PCR-free-150pM.clean.dedup.recal.bam.recalibrated.snp.annotated.vcf`

NA11992 `https://export.uppmax.uu.se/a2009002/opendata/HiSeqX_CEPH/CEP-NA11992/04-VCF/CEP-NA11992.clean.dedup.recal.bam.recalibrated.indel.annotated.vcf` and `https://export.uppmax.uu.se/a2009002/opendata/HiSeqX_CEPH/CEP-NA11992/04-VCF/CEP-NA11992.clean.dedup.recal.bam.recalibrated.snp.annotated.vcf`

Other Platinum Genome VCFs were obrained from dbGaP (project phs001224). For PGRNseq samples, we obtained VCF files with GATK Best Practices pipeline.

Astrolabe (version v0.7.5) was run as follows:

```
astrolabe-0.7.5/run-astrolabe.sh \
  -inputVCF sample.vcf.gz \
  -inputBam sample.bam \
  -conf astrolabe-0.7.5/astrolabe.ini \
  -outFile sample.log
```

The inputBam parameter was not used for samples NA11832, NA19238, NA19239, NA19900 and NA19240.

5.4 PGRNseq v.2 validations

Samples in the PGRNseq v.2 dataset were tested for the following *CYP2D6* SNPs: rs16947, rs35742686, rs1800716, rs5030866, rs5030867, rs5030656, rs1065852, rs59421388, rs28371706 and rs28371725. They were

also tested for the presence of duplications, whole gene deletions and *CYP2D7/2D6* hybrids by XL-PCR. This data was generated by Andrea Gaedigk's lab (Kansas City Mercy Hospital and University of Missouri-Kansas City).

References

[1] Engelbeen, C., Fiorini, S. & Kiesel, A. A closest vector problem arising in radiation therapy planning. *Journal of combinatorial optimization* **22**, 609–629 (2011).

[2] Pratt, V. M. *et al.* Characterization of 137 Genomic DNA Reference Materials for 28 Pharmacogenetic Genes: A GeT-RM Collaborative Project. *The Journal of molecular diagnostics: JMD* **18**, 109–123 (2016).

[3] Riffel, A. K. *et al.* CYP2D7 Sequence Variation Interferes with TaqMan CYP2D6 (*) 15 and (*) 35 Genotyping. *Frontiers in Pharmacology* **6**, 312 (2015).

[4] Fang, H. *et al.* Establishment of CYP2D6 reference samples by multiple validated genotyping platforms. *The pharmacogenomics journal* **14**, 564–572 (2014).
